# Rhabdomyolysis and Hyperosmotic Hyperglycemic State as the Initial Presentation of Type II Diabetes Mellitus in an Adult Male: A Case Report

**DOI:** 10.51894/001c.165617

**Published:** 2026-07-31

**Authors:** Jessica Mills, Muhammad Ahmed, Fnu Rashi, John Polyzois, Nada Al-Antary, Ghada Mesleh

**Affiliations:** 1 Henry Ford - Macomb

## 102

### Introduction

Hyperosmolar hyperglycemic state (HHS) with confounding rhabdomyolysis as the initial manifestation of type 2 diabetes mellitus in young adults is exceedingly rare. While HHS is a well-recognized complication of poorly controlled diabetes, rhabdomyolysis in association with HHS is uncommon and further complicates the clinical presentation. HHS is a severe metabolic complication of diabetes mellitus characterized by profound hyperglycemia, hyperosmolarity, and marked dehydration with minimal or absent ketoacidosis. It most frequently occurs in older adults with symptoms of hyperglycemia and dehydration, including polyuria, polydipsia, and altered mental status. Presentation in younger individuals without a prior diagnosis of diabetes is unusual, and neuromuscular symptoms such as proximal lower extremity weakness are atypical presenting features.

### Case Presentation

We present the case of a 31-year-old male with no significant past medical history who presented with progressive bilateral lower extremity weakness. Initial evaluation revealed profound hyperglycemia (serum glucose 1517 mg/dL) and elevated calculated serum osmolality (398 mOsm/kg), consistent with HHS. Arterial blood gas demonstrated mild acidosis (pH of 7.34). Additional laboratory studies revealed an elevated creatine kinase level of 6092 IU/L, consistent with rhabdomyolysis, and evidence of acute kidney injury and electrolyte abnormalities. MRI lumbar and thoracic spine were unremarkable. The patient denied recent trauma, prolonged immobilization, seizures, substance use, or medication exposure that could otherwise account for rhabdomyolysis. Management included aggressive intravenous fluid resuscitation, continuous insulin infusion, and monitoring for correction of electrolyte abnormalities. Creatine kinase levels progressively declined with treatment, and renal function improved during hospitalization.

### Discussion

Rhabdomyolysis is an uncommon but important complication of HHS and may be underrecognized due to overlapping clinical features with severe dehydration and renal dysfunction. Although the mechanism remains incompletely understood, proposed causes include direct skeletal muscle injury from hyperosmolarity, decreased muscle perfusion due to profound intravascular volume depletion, and electrolyte abnormalities. Early identification is critical, as prompt fluid resuscitation and metabolic correction prevent severe renal injury. This case underscores the importance of maintaining suspicion for rhabdomyolysis in HHS, particularly with unexplained muscle weakness or elevated creatine kinase levels. Recognition of this rare association may facilitate earlier diagnosis and improve outcomes.

